# Interaction of Complement Factor H and Fibulin3 in Age-Related Macular Degeneration

**DOI:** 10.1371/journal.pone.0068088

**Published:** 2013-06-28

**Authors:** M. Keith Wyatt, Jen-Yue Tsai, Sanghamitra Mishra, Maria Campos, Cynthia Jaworski, Robert N. Fariss, Steven L. Bernstein, Graeme Wistow

**Affiliations:** 1 Section on Molecular Structure and Functional Genomics, National Eye Institute, National Institutes of Health, Bethesda, Maryland, United States of America; 2 Biological Imaging Core, National Eye Institute, National Institutes of Health, Bethesda, Maryland, United States of America; 3 Laboratory of Retinal Cell and Molecular Biology, National Eye Institute, National Institutes of Health, Bethesda, Maryland, United States of America; 4 Departments of Ophthalmology and Neurobiology & Genetics, University of Maryland School of Medicine, Baltimore, Maryland, United States of America; Eye Hospital, Charité, Germany

## Abstract

Age-related macular degeneration (AMD) is a major cause of vision loss. It is associated with development of characteristic plaque-like deposits (soft drusen) in Bruch’s membrane basal to the retinal pigment epithelium (RPE). A sequence variant (Y402H) in short consensus repeat domain 7 (SCR7) of complement factor H (CFH) is associated with risk for “dry” AMD. We asked whether the eye-targeting of this disease might be related to specific interactions of CFH SCR7 with proteins expressed in the aging human RPE/choroid that could contribute to protein deposition in drusen. Yeast 2-hybrid (Y2H) screens of a retinal pigment epithelium/choroid library derived from aged donors using CFH SCR7 baits detected an interaction with EFEMP1/Fibulin 3 (Fib3), which is the locus for an inherited macular degeneration and also accumulates basal to macular RPE in AMD. The CFH/Fib3 interaction was validated by co-immunoprecipitation of native proteins. Quantitative Y2H and ELISA assays with different recombinant protein constructs both demonstrated higher affinity for Fib3 for the disease-related CFH 402H variant. Immuno-labeling revealed colocalization of CFH and Fib3 in globular deposits within cholesterol-rich domains in soft drusen in two AMD donors homozygous for CFH 402H (H/H). This pattern of labeling was quite distinct from those seen in examples of eyes with Y/Y and H/Y genotypes. The CFH 402H/Fib3 interaction could contribute to the development of pathological aggregates in soft drusen in some patients and as such might provide a target for therapeutic intervention in some forms of AMD.

## Introduction

Age related macular degeneration (AMD) is a major cause of vision loss in aging western populations [1]. AMD may manifest in different ways, for example in “dry” or “wet” (with neovascularization) forms [2], [3]. The disease has been associated with light-induced oxidative damage [4], [5], accumulation of cholesterol and other lipids [6], and has been linked to systemic factors such as smoking, hypertension and atherosclerosis [7], [8], [9], [10]. A recent study has shown loss of activity of the enzyme Dicer1 and toxicity from accumulating *Alu* RNA in the terminal stage of AMD [11], however many different upstream events may lead to this final outcome. Furthermore, the growing list of genes associated with AMD risk, coupled with the recognition that there are multiple pathological phenotypes under the broad classification of AMD shows this may be a family of distinct diseases with a common outcome.

AMD is generally associated with pathological changes in the retinal pigment epithelium (RPE) and Bruch’s membrane (a collagen-rich extracellular matrix between the RPE and choroidal vasculature) including formation of plaque-like deposits called drusen [12]. Drusen are often classified as “hard” or “soft” (also called “large”) and while the hard form is common in the aging human eye, soft drusen are more closely associated with risk and progression of AMD [13]. Proteomics and immuno-labeling studies have shown that drusen of all kinds contain a number of proteins, including several components of the complement system, and cholesterol-rich lipids [6], [14], [15], [16].

Linkage analyses and candidate gene approaches have found significant association of AMD with sequence variants in the genes for complement factor H (CFH) [17], [18], [19], [20] and the CFH-related gene cluster [21], [22] on human chromosome 1 and with the gene for the serine protease HTRA1 [23] or the neighboring *LOC387715* [24] on chromosome 10. Associations with several other factors, including complement factors B, C2 [25], C3 [26] and D, and others such as the chemokine receptor CX3CR1 [27], have supported a general inflammatory connection to AMD, although a growing number of other genes have also been linked to the disease, suggesting a degree of heterogeneity [28], [29].

In spite of this genetic diversity, possession of a common polymorphic variant (402Y>H) in CFH has been associated with as much as half the risk of developing the dry form of AMD [17] in Caucasian populations. CFH is a major regulator of complement activation through the alternative pathway (AP) [30]. The protein consists of 20 short consensus repeat (SCR) domains and has several binding sites for proteins such as complement component 3b (C3b) as well as for bacterial cell wall components, heparin and other ligands [30]. CFH dependent cleavage of C3b acts to shut down the cascade of complement activation, thereby localizing its cytolytic and inflammatory effects [30]. CFH also binds the inflammatory C-reactive protein (CRP) and elevated levels of CRP in RPE/choroid have been reported in AMD [31], [32]. Two-year old CFH null mice exhibit retinal and RPE problems, suggesting that loss of this regulatory protein can damage the eye [33], although the defects in these mice do not closely resemble AMD. CFH deficiency in humans is associated with kidney lesions in membranoproliferative glomerulonephritis, type II (MPGN II; OMIM 609814). MPGN II patients have been reported to have abundant drusen [34], but AMD itself is not associated with the condition or with kidney disease. Recent studies have suggested an interplay among variants in CFH itself and the CFH-related gene cluster, with both protective and risk associations [22], [35].

Given the particular association of CFH variants with AMD, it is possible that this protein is capable of specific binding interactions with components of the aging RPE/choroid itself, leading either to dysregulation of complement or to formation of pathological protein aggregates at Bruch’s membrane. To investigate possible binding targets for the disease risk domain SCR7 of CFH, we undertook a yeast 2-hybrid screen of expressed cDNAs from RPE/choroid from aged donors and identified a potential partner which also has associations with macular degeneration.

## Methods

### Yeast 2-hybrid (Y2H)

A custom Y2H cDNA library was constructed from pooled RPE/choroid from five donors, males and females, 51–75 years of age using the pGADT7-rec vector in the MatchMaker System (Clontech, Mountain View, CA). Post-mortem tissue was obtained under University of Maryland Institutional Review Board (IRB) exemptions SB-019701 and SB-129901. The unamplified library had 5×10^7^ independent cDNA clones with an average insert size of 1.1 kbp.

Baits for CFH SCR domain 7 were constructed by PCR of cDNA clones and genomic DNA using primers CFHD7.NDE: CATATGAAATGTTATTTTCCTTATTTG; CFHD7.BAM: GGATCCTATTTGACACGGATGCATCTGGG. Variants were identified by sequencing, cloned into the pGBKT7-rec bait vector and transformed into yeast strain Y187.

Expression of CFH bait fusion proteins was validated by Western blot using GAL4 DNA-BD monoclonal antibody (Clontech).

Approximately 10^7^ bait yeast were mated with approximately 10^6^ prey (library) yeast following protocols in the Clontech Yeast Protocols Handbook (PT3024-1). Interacting colonies were selected by growth on 4DO (drop out) (-Ade, -His, -Leu, -Trp) medium for up to 4 days. Colonies were tested for β-galactosidase (βGal) activity and for self activation. Validated clone plasmids were isolated using the Wizard Plus SV system (Promega, Madison WI) and sequenced by standard methods. Two additional preys, FIB3 EGF and Fib3 CT were constructed by PCR of the Fib3 parent plasmid using primers:

FIBEGFNDE: CATATGGGCAGGCTCAACTGTGAAGACATTG;

FIBEGFBAM: GGATCCTCAAACACATCGGTTCTCTGGTGTT;

FIBCTNDE: CATATGCCAGTCTCAAATGCCATGTGCCGAG;

FIBCTBAM: GGATCCCTAAAATGAAAATGGCCCCACTATT.

These preys were also tested against CFH-H bait for growth on 4DO medium and tested for α-galactosidase activity.

### Quantitative βGal Assay

Five individual clones for CFH H402 (H) or Y402 (Y) bait constructs were mated to Fib3 bait and assayed for βGal activity in liquid culture using CPRG (chlorophenyl red-β-D-galactopyranoside) substrate (Yeast Protocols Handbook (PT3024-1), Clontech). OD_578_ within the linear range (above 0.25) was measured and activity was expressed in Miller Units: ( = 1000×OD_578_/t×V×OD_600_, where t = incubation time in min; V = 0.1 concentration factor).

### Recombinant Proteins

The HAFib3 expression construct was made by PCR of Y2H prey plasmid using primers: NHEpGAD: GCTAGCATGGAGTACCCATACGACGTACCAG; XHOpGAD: CTCGAGCTCGATGGATCCCGTATCGATG. This was cloned into NheI/XhoI sites of pET17b (Novagen, Madison WI) and expressed in *E.coli* Ril (Stratagene, La Jolla, CA). Protein was prepared from inclusion bodies, denatured, refolded [36] and purified with an HA affinity column.

Recombinant protein for CFH domains 6–8 (CFH678) was expressed in *Pichia pastoris*, using the pPICZαA expression vector (Invitrogen, Carlsbad, CA) [37]. The CFH678 sequence was derived by PCR from a cDNA clone corresponding to the 402H variant using primers: 5CFH68pz: GAATTCTTGAAACCTTGTGATTATCC; 3CFH68pz: TCTAGATTTAATGCACGTGGGTTGAGC and cloned using EcoRI/XbaI sites. The 402Y variant was engineered by site specific mutagenesis (GenWiz, South Plainfield, NJ). Secreted recombinant protein was purified using the AKTA Explorer system (GE Healthcare).

### ELISA

The concentrations of purified recombinant proteins were determined by bicinchoninic acid assay (Pierce). 96-well microtiter plates were coated with mouse anti-HA (Invitrogen) 5 ng/µl in 50 mM sodium carbonate pH 8.8 overnight at 4°C. Plates were blocked with BSA, 5% in sodium carbonate, 50 mM, pH 8.8 for 3 hr at room temperature then incubated with HAFib3 250 ng/ml in 25 mM Tris, 150 mM NaCl, pH 7.4 overnight at 4°C followed by washing with PBS/Tween 20, 0.05%. Control wells had no added HAFib3. A range of concentrations of CFH678 (H or Y) in PBS was added and incubated overnight at 4°C, followed by washes with PBS/Tween, 0.05% at RT. Interaction was detected by adding goat anti-CFH antibody (1∶5000) for 1 hr RT. After washing, HRP-labeled secondary antibody (1∶40,000) was added, incubated for 1 hr and washed. HRP substrate (SureBlue, KPL), 100 µl/well was added and incubated until a blue color in a measurable range appeared. Reactions were quenched with 0.6 N sulfuric acid and read on Victor Multilabel counter (Wallac) at 450 nm. Background for control wells lacking HAFib3 was subtracted from experimental values for 8 replicates.

Non-linear regression analysis was performed on the ELISA binding data, using the algorithm for one –site specific binding in GraphPad Prism version 5.03 for Windows (GraphPad Software, San Diego CA).

### Cell Culture

ARPE-19 cells [38] were grown in DMEM/Ham’s F12 50/50, 1.5 mM L-Glutamine, 0.8 mM sodium pyruvate, 0.08 mM non-essential amino acids, 0.1% penicillin/streptomycin, 10% FBS. HEK 293 cells were grown in DMEM containing 10% FBS, 1% penicillin/streptomycin. Reagents were purchased from Gibco BRL (Carlsbad, CA).

ARPE-19 cells of passage number 10–20 were grown in 6 well plates in prescribed growth medium under 5% CO2, 37°C. At 70% confluence, cells were washed twice and kept in serum free medium (DMEM, 1% penicillin/streptomycin). Cells achieved confluence under serum-free conditions. Cells were checked for RPE phenotype by morphological examination and RT-PCR detection of marker transcripts RPE65 and CRALBP [38].

For immunoprecipitation, CFH (Complement Technology, Inc., Tyler, TX) at 0, 2 or 5 µg/ml was added on day 5 of serum deprivation.

### Immunoprecipitation

Antibodies used were goat polyclonal anti-human CFH, horse radish peroxidase (HRP) conjugated polyclonal donkey anti-goat IgG (Abcam Inc., Cambridge, MA), goat anti-rabbit IgG-HRP (Bio-Rad Laboratories, Hercules, CA). A new anti Fib3 antibody was raised in rabbits immunized against peptide NNEQPQQETQPAEGTSG (21st Century Biochemicals, Inc., Marlboro, MA). For verification, this antibody was compared with the previously described mouse monoclonal [39] (gift from Dr. Lihua Marmorstein) in Western blot of ARPE-19 conditioned medium and of a Western blot control recombinant GST-Fib3 fusion protein expressed in wheat germ extract purchased from Abnova (Taipei, Taiwan). Identical results were obtained as shown in Figure S1.

Sulfo-NHS-LC-Biotin and Streptavidin Magnetic beads (Pierce Biotechnology, Inc. Rockford, IL) were diluted to 1 mg/1 ml in PBS, washed 3×5 min in PBS followed by equilibration in DMEM, 2×5 min. 1 mg primary antibody was labeled with 13µl of 10 mM Biotin on ice for 2 hours then dialyzed against PBS at 4°C overnight.

Conditioned medium was pre-cleared with 30µl of 1 mg/ml DMEM equilibrated streptavidin magnetic beads for 30 min, prior to immunoprecipitation with 5µg of biotinylated antibodies, overnight at 4°C. 100µl 1 mg/ml DMEM equilibrated Streptavidin magnetic beads were added, incubated for 2 h, collected using Magnetic Separation Rack (New England Biolabs, Ipswich, MA), washed 3x with PBS and incubated in 50 µl 2X SDS loading buffer at 42°C for 30 min. Proteins resolved by SDS PAGE were transferred to PVDF membrane followed by Western blotting using goat polyclonal anti-CFH (1∶3000).

The reciprocal experiment was performed using day 7 ARPE-19 medium with and without the addition of 2µg human CFH and using biotinylated antibody to human CFH. Western blot with rabbit antibody to Fib3 detected Co-IP of Fib3.

### Genotyping Donor Eyes

Normal and AMD-affected human donor eyes fixed in formalin were obtained from the National Disease Research Interchange (NDRI), Philadelphia, PA under NIH OHSR exemption 11287. Genomic DNA was prepared from fixed eye tissue using MagneSil Genomic, Fixed Tissue System, (Promega, Madison WI). Polymerase chain reaction was performed using HotStar HiFidelity system, (QIAGEN, Valencia, CA), with primers specific to the CFH gene (CFHgenS: ATCTTATTTAAATATTGTAAAAATAATTGT, CFHgenAS: TAAACATTTTGCCACAATTAATATAGATG). The amplified 375 bp product, spanning the Y402H region of the CFH gene, was purified from the PCR reaction using Qiaquick PCR purification kit, (QIAGEN) and sequenced at ACGT Inc. (Wheeling, IL) using sequencing primers CFHPCRS: ACTATTTTGAGCAAATTTATG; CFHPCRAS: GTCTTAGAATGTCATCTATG. Sequences and traces were examined using SeqMan (DNASTAR, Madison, WI).

### Immunofluorescence

Normal and AMD-affected human donor eyes from NDRI were formalin fixed, washed in PBS and cryoprotected in PBS/sucrose. Eyes were cut and sectioned through the macula (Fig. S2A). 7µm cryosections were collected on Superfrost (plus) slides. Sections were incubated with ICC buffer (0.5% BSA, 0.2% Tween 20, 0.05% sodium azide, in PBS, pH 7.3) for 30 minutes, labeled with primary antibodies for 1 hour at RT, washed with ICC buffer, labeled with secondary antibodies and DAPI (Invitrogen) for 30 minutes at RT, washed extensively with ICC buffer, mounted and imaged with Leica SP2 confocal microscope (Exton, PA). Primary antibodies were goat anti-human CFH (1∶200) (Quidel, San Diego CA), mouse anti-Fib3 [39] (1∶100), rabbit F1-1 anti-Fib3 (1∶200), mouse anti-rhodopsin ID4 (1∶100) (Chemicon, Temecula, CA) and rabbit anti-GFAP (1∶300) (DAKO, Carpinteria, CA). Secondary antibodies were Alexa 488 donkey anti-rabbit, Alexa 555 donkey anti-mouse and Alexa 633 donkey anti-goat (Invitrogen). For several figures, the autofluorescence signal from lipofuscin was collected with 488 nm laser excitation and 580–650 nm emission bandwidth.

One figure used biotinylated Peanut Agglutinin (Vector labs) (1∶500) detected with streptavidin conjugated with Alexa 633(Vector labs) (1∶300); mouse rhodopsin MAB5356 (Millipore) (1∶100) with secondary Alexa488); rabbit polyclonal against RPE65 (1∶100) (T. M. Redmond, National Eye Institute) with secondary Alexa 555.

Filipin (Sigma) 0.05 mg/ml in PBS was used to label unesterified cholesterol. Sections were incubated with 1 ml of filipin for 2 hours at RT in the dark and washed 3X with PBS. When using filipin, TO-PRO-3 (T3605 642/662 Invitrogen, 1∶500 dilutions) was as a nuclear stain.

Adjacent cryosections were also used for hematoxylin and eosin (H&E) staining to illustrate histology. 10 µm sections were held under vacuum overnight, dipped 3 times in 70% ethanol and rinsed in distilled water. They were stained with Gill’s haematoxylin (Vector Labs) for 5 minutes, rinsed in water, dipped three times in ammonia solution, rinsed and stained with Eosin Y (Baker Analyzed) for 3 minutes, dehydrated in 5 quick dips in 2 changes of 95% ethanol and 2 changes of 100% ethanol then 10 dips in 2 changes of xylene, cleared and mounted using VectaMount H-5000 (Vector Labs) and imaged using a BX51 Olympus microscope.

## Results

### Yeast 2-Hybrid (Y2H) Screening

Yeast 2-hybrid screens provide a method for searching for potential interactions of a protein domain with the expressed proteome of a target tissue. [40]. A custom Y2H cDNA library was constructed from pooled RPE/choroid from aged Caucasian donors. Baits were made for CFH SCR7, containing either Y or H at position 402 and for the equivalent domain of the closely related *CFHR3* gene (which is also associated with AMD [21]) and were independently screened against the RPE/choroid library. A total of 160 colonies that grew on selective medium were selected for verification, checking for βGal activity, self-activation and ability for pairwise interaction with baits. After elimination of false positives and self-activating prey clones, 40 validated clones were obtained and were sequenced. Some corresponded to artifactual open reading frames from non-coding sequence or were partial cDNA clones that incorporated intron sequence. 14 clones contained spliced cDNAs for genes for a variety of intracellular, membrane and secreted factors (Table 1). Several of these potentially relevant interactors corresponded to membrane or secreted proteins. One potential binding partner for CFH, the extracellular matrix (ECM) protein Fibulin3 (Fib3), also known as epidermal growth factor-containing fibulin-like extracellular matrix protein 1(EFEMP1), stood out as a protein with increased deposition basal to RPE in AMD [39].

**Table 1:** Validated prey clones from Y2H screen with CFH SCR7 domain baits.

Gene	Compartment	Function	Domain
C1QA	secreted	complement	Collagen/C1q
CSF1R	plasma membrane	proliferation	Ig domains
DCAF7	intracellular	signaling	WD repeat
EFEMP1/Fib3	secreted	ECM	EGF-like
FCGBP	secreted	IgG binding	VWD domain
LAM5A	secreted	ECM	EGF-like
Notch 3	plasma membrane	signaling	EGF-like
PCBP1	intracellular	RNA binding	KH-like domain
PLXNB2	plasma membrane	signaling	Plexin repeat
PMEL	intracellular	melanin biosynthesis	PKD domain
PSAP	secreted/lysozome	lipid binding	Saposin domain
PTPRJ	plasma membrane	signaling	Fibronectin 3 domain
TRAF3IP2	intracellular	signaling	SEFIR domain
TUBB4B	intracellular	cytoskeleton	β-tubulin

Compartment, function and domain information are taken from Entrez Gene http://www.ncbi.nlm.nih.gov/gene/.

**Figure 1:**
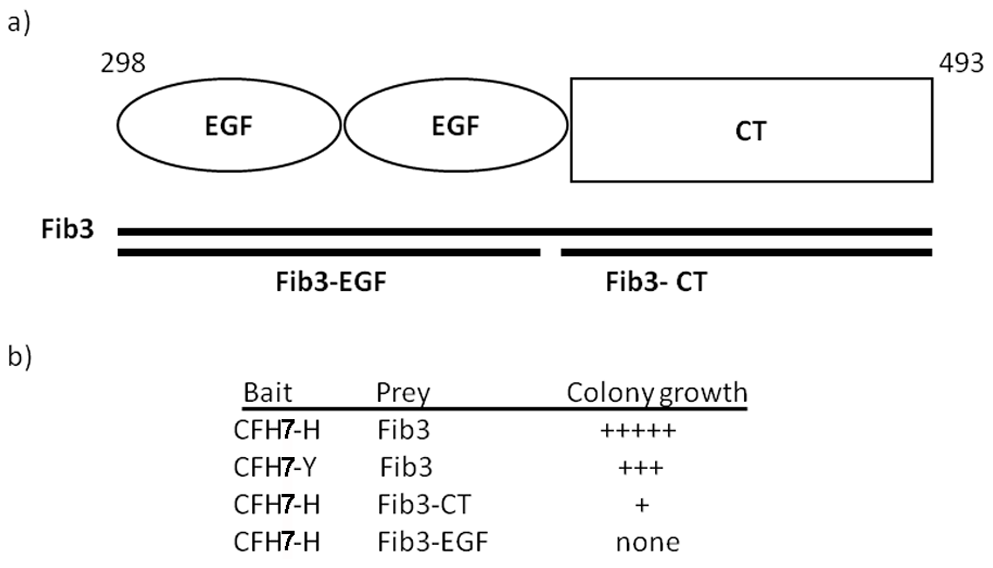
CFH SCR7 baits bind Fib3 prey in yeast 2-hybrid (Y2H) assays. **a)** Cartoon of the Fib3 prey clone, corresponding to amino acid residues 289–493. EGF-like and C-terminal (CT) domains are indicated. Bars indicate the Fib3-EGF and Fib3-CT domains use to localize the CFH interaction site. **b)** Tabulation of relative colony growth on selective medium (4DO) for bait/prey combinations.

The Fib3 prey fusion protein from the Y2H screen consists of two EGF-like domains and the C-terminal domain of the protein [41] (Fig. 1a). It interacted with both H and Y variants of the CFH SCR7 bait, giving colony growth and βGal expression on high stringency (4DO) medium. Interestingly, the disease associated CFH-H bait gave qualitatively the most abundant and rapid colony growth, consistent with a stronger interaction (Fig. 1b).

To localize the site of interaction for CFH SCR7 on Fib3, separate prey constructs were made for the pair of EGF-like domains and for the C-terminal domain. The CFH-H bait interacted with the original Fib3 prey and with the C-terminal domain, although the interaction was qualitatively weaker than for the original Fib3 prey clone, but failed to interact with the EGF domains (Fig. 1b). This suggests that the site of interaction for CFH and Fib 3 is in the C-terminal domain. This is also the site of interaction of Fib3 with another ECM protein, TIMP3 [42] which itself is mutated in Sorsby fundus dystrophy, another inherited disease which has similarities to macular degeneration [43], and is also associated with AMD [28].

### Co-immunoprecipitation of Native CFH and Fib3

**Figure 2:**
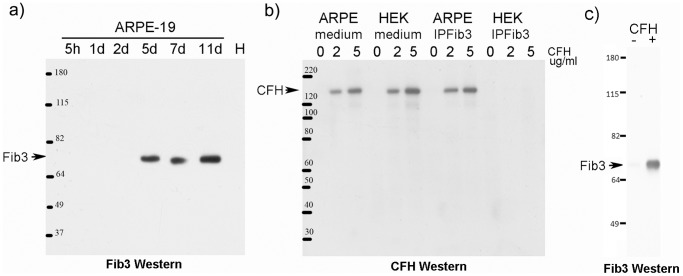
Interaction of native CFH and Fib3. **a) Fib3 expression is induced in serum-starved ARPE-19 cells in culture.** Western blot of serum-starved conditioned medium with anti-Fib3 antibody. For ARPE-19 cells, Fib3 is induced after 5 days in culture. No Fib3 is detectable in HEK293 cell conditioned medium after 7 days under the same conditions (H). **b) Co-immunoprecipitation of native CFH in presence of Fib3.** ARPE-19 (ARPE) or HEK 293 (HEK) cells were serum deprived for 5 days followed by addition of 0, 2 or 5 µg/ml of purified human Complement Factor. After 48 h conditioned media from each sample were subjected to immunoprecipitation with anti-Fib-3.ARPE and HEK lanes on the left show Western blot of media before IP using anti-CFH. Lanes on the right show western blots after immunoprecipitation. Co-IP of CFH with Fib3 antibody occurs only in ARPE-19 medium which contains Fib3 (as shown in Fig. 2a). c) **Co-immunoprecipitation of native Fib3 in presence of CFH.** Medium from 7 day serum starved ARPE-19 cells with or without addition of 2µg human CFH was immunoprecipitated with antibody to CFH and Fib3 was detected by Western blot.

Results from Y2H showed that the single CFH SCR7 domain can interact with Fib3 in yeast, so we asked whether the native human CFH and Fib3 proteins can interact in solution. ARPE-19 cells [38] have been reported as a source of secreted Fib3 [39] so we used conditioned medium from these cells to confirm the CFH/Fib3 interaction. Cells of moderate passage number (10–20), positive for expression of RPE markers RPE65 and CRALBP [38], were grown to near confluence and were then transferred to serum-free medium to remove bovine serum components. Initially Fib3 was undetectable by Western blot in conditioned medium, but after 5 days in the absence of serum, a significant level of secreted Fib3 was detected (Fig. 2a). Another cell line, HEK293, was treated identically but showed no detectable Fib3 expression up to 7 days. Conditioned media from these two cell types after 7 days in culture were treated with 0, 2 or 5µg of commercial purified human CFH (from pooled serum) and immunoprecipitation (IP) was performed using antibody to Fib3 (Fig. 2b). Western blots for the Fib3 IP showed the presence of co-IP CFH only in the ARPE-19 samples with added CFH, consistent with an interaction of native CFH and Fib3 in solution. Similarly, day 7 ARPE-19 medium, with or without the addition of 2µg CFH was immunoprecipitated with goat anti-CFH antibody. Western blots with rabbit antibody to Fib3 showed a strong band for Fib3 in the presence of added CFH (Fig. 2c). This shows that native human CFH and Fib3 can interact in solution.

### Binding of CFH Variants and Fib3 by Quantitative Y2H

**Figure 3:**
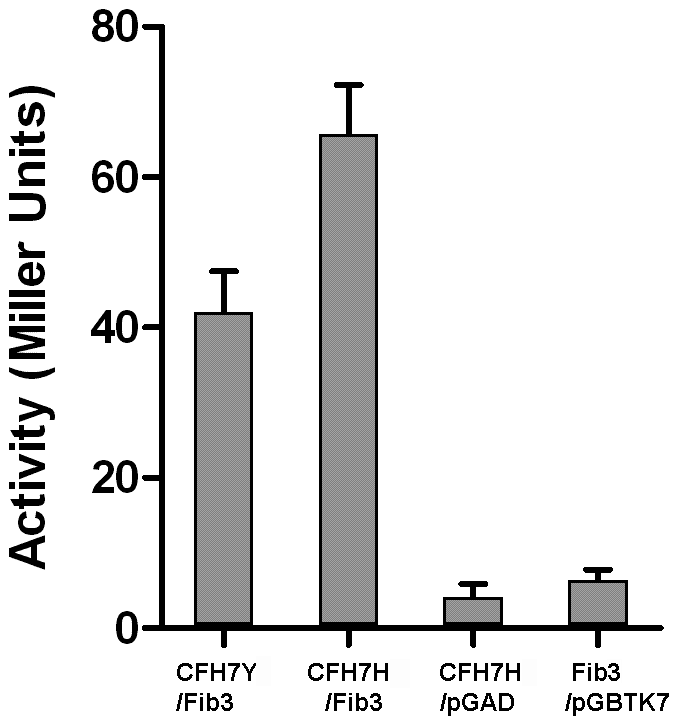
CFH SCR7 402H variant binds Fib3 with higher affinity than the Y variant in Y2H. Yeast clones containing the Fib3 prey construct and either the CFH 402H (CFH7H) or 402Y (CFH7Y) bait constructs were tested in quantitative β-galactosidase assay. Controls shown are CFH7H bait with empty prey vector (pGAD) and Fib3 prey with empty bait vector (pGBTK7). Activity is expressed in Miller Units: ( = 1000×OD_578_/t×V×OD_600_, where t = incubation time in min; V = 0.1 concentration factor). Standard deviation error bars (N = 8) are shown.

We next asked whether the CFH SCR7 variants showed differences in Fib3 binding. Since interaction between bait and prey fusion proteins in Y2H leads to activation of βGal expression in the yeast host, the relative strength of interaction can be measured in terms of normalized βGal activity [44]. Five independent yeast clones containing either the CFH-H or CFH-Y SCR7 baits together with the original Fib3 prey clone were tested for βGal activity in selective liquid culture. Both bait/prey combinations activated βGal but the disease-associated CFH-H variant gave 60% higher activation than CFH-Y (Fig. 3), consistent with a higher affinity interaction with the Fib3 prey.

### Binding of Recombinant CFH Variants and Fib3 by ELISA

**Figure 4:**
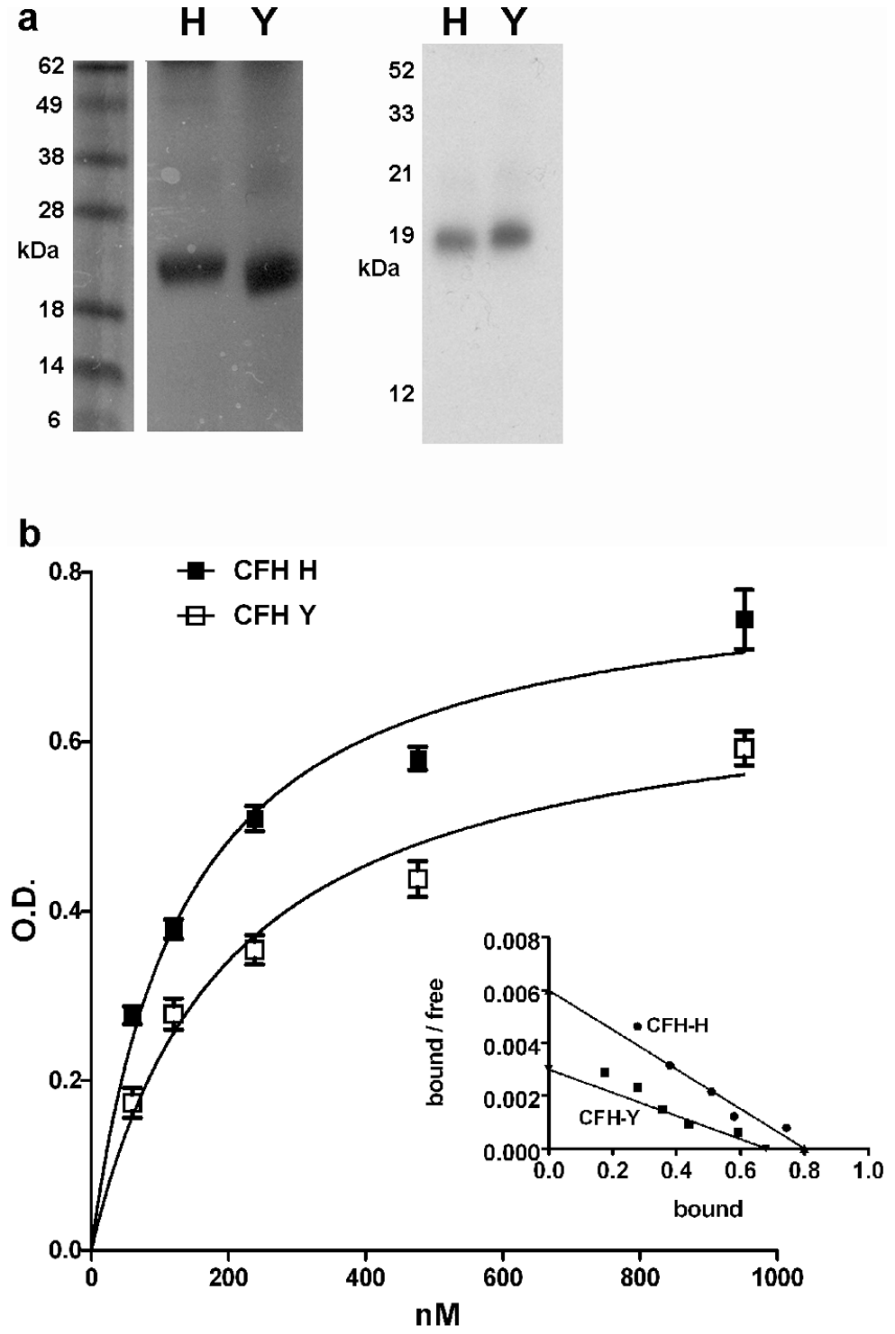
ELISA for recombinant constructs for CFH and Fib3 shows higher affinity binding for the CFH-H variant. a) Recombinant CFH678 constructs. Left: Coomassie stain for H and Y variants. Right: Western blot of constructs using goat anti-human CFH antibody. b) ELISA. Anti-HA antibody was anchored to the plate and used to capture HAFib3. Concentration ranges of CFH678 H and Y variants were added and detected with anti-CFH antibody, visualized with fluorescent labeled secondary antibody at measured at 450 nm. Measurements were replicated 8-fold and background for each point (ELISA with no added HAFib3) was subtracted. Curves were calculated by non-linear regression. Standard error of mean (SEM) is indicated for each point. The Scatchard plot for the same data is shown as an insert. Kd: CFH-H = 134 nM +/−16); CFH-Y: = 197 nM +/−29. Bmax: CFH-H = 0.8+/−0.03; CFH-Y = 0.7+/−0.03 (in OD units).

We next compared the Fib3 affinity of the CFH variants *in vitro* using recombinant protein constructs. Recent studies on CFH binding activity have used a partial recombinant CFH protein, consisting of SCR domains 6–8 (CFH678) expressed in *Pichia pastoris* [37] and we adopted this established approach for both H and Y variants (Fig. 4a). This allowed both variants to be produced under identical conditions in the absence of other human serum proteins (as opposed to fractionation from whole human serum). For Fib3, to produce an N-terminal tag distant from the C-terminal binding site, a construct consisting of the Fib3 prey fragment (two EGF domains and the C-terminal domain) with an N-terminal hemagglutinin tag (HAFib3) was expressed in *E.coli*, refolded and purified using an HA affinity column.

These recombinant proteins were used in ELISA. Anti-HA antibody was bound to support and used to capture HAFib3 after which CFH678 proteins (H and Y variants) were added and detected using anti-CFH antibody (which had been verified for binding to the CFH678 construct) and secondary antibody. As shown in Figure 4b, both CFH-H and Y variant constructs bound HAFib3 in a concentration dependent manner. Non-linear regression analysis showed a higher affinity for the interaction with the H variant (CFH678-H: Kd = 134 nM +/−16(SEM); CFH678-Y: Kd = 197 nM +/−29 (SEM). Thus both isolated CFH SCR7 in yeast and the recombinant SCR6–8 in ELISA demonstrate binding to Fib3 targets and in both cases the disease-associated 402H variant shows higher affinity binding.

### Co-localization of CFH and Fib3 in AMD Eye

**Figure 5:**
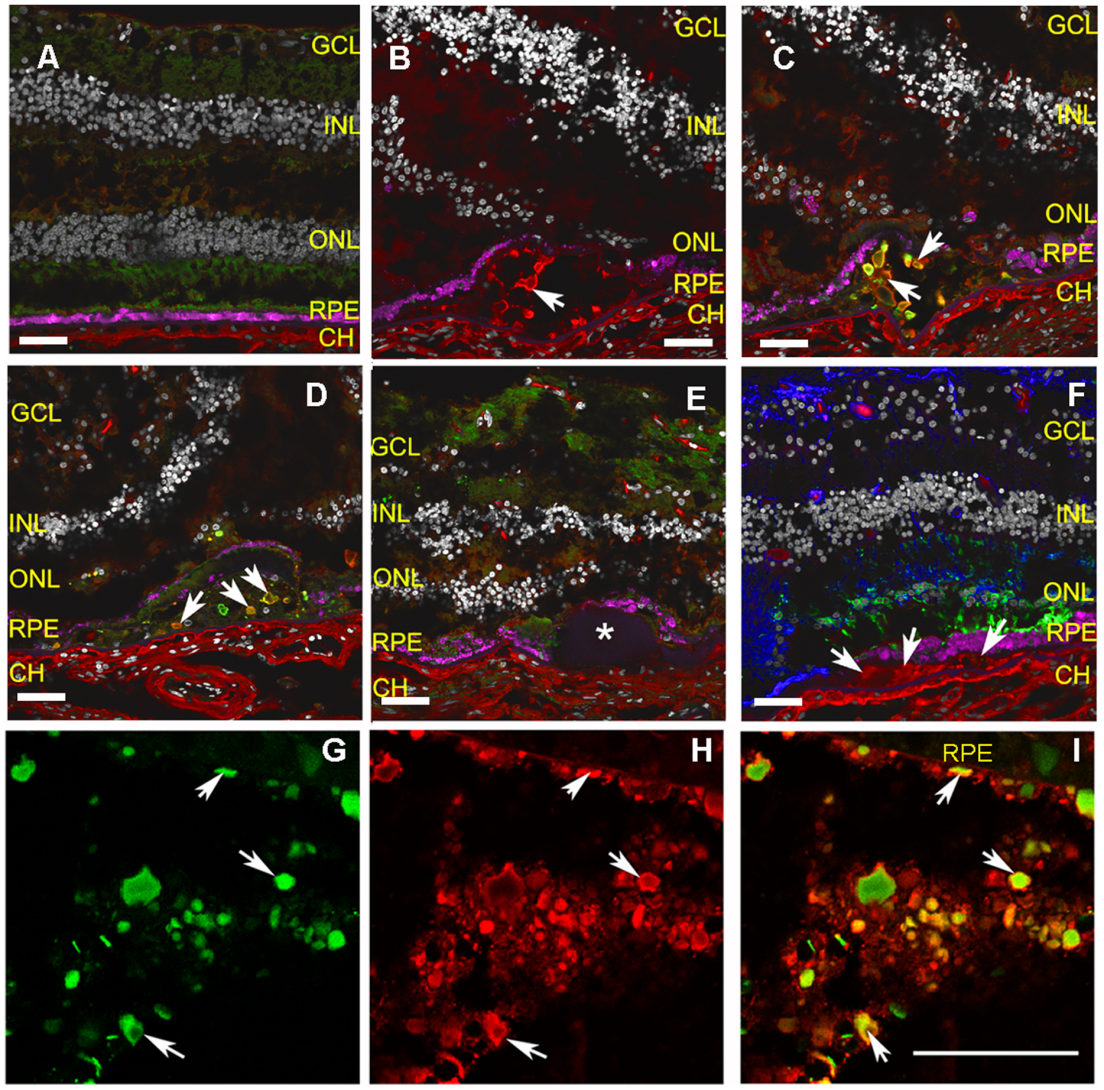
Immunofluorescence of CFH, Fib3 and controls in normal and dry AMD eye GCL (ganglion cell layer), INL (inner nuclear layer), ONL (outer nuclear layer), RPE (retinal pigment epithelium), CH (choroid). Nuclei (gray) and autofluorescence from lipofuscin granules (magenta) are visible in all cryosections. Scale bar = 50µM. **A:** Section of retina from a normal human eye labeled with mouse monoclonal antibody against Fib3, mFib3 (green) and an anti-CFH antibody (red). In normal retina, the Fib3 immunoreactivity is present in the ganglion cell layer as reported previously. CFH is restricted to choroidal blood vessels. **B**: Section from H/H AMD eye (#57985) with soft druse. CFH (red) is detected in globules within the druse. **C:** Similar section to B labeled for both mFib3 (green) and an anti-CFH antibody (red). Strong Fib3 immunoreactivity is observed in the soft druse (arrows) and the signal colocalizes with that of anti-CFH. Merged colors vary according to relative staining for both antibodies. **D:** The colocalization of Fib3 and CFH in soft drusen (arrows) is confirmed with a newly generated anti-fib3 antibody-rFib3 (rabbit polyclonal antibody raised against the N-terminal region of human Fib3 (blue). The two independently derived anti-Fib3 antibodies show identical staining patterns. (See also Fig. S3). **E:** Little labeling of either Fib3 or CFH was observed in a hard druse (*) from the same AMD donor (left panel). **F:** Control antibodies for markers for retinal glia (anti-GFAP rabbit polyclonal, blue) and rod photoreceptors (anti-rhodopsin mouse monoclonal, green) do not label CFH-positive areas within the soft drusen (arrows). **G–I:** Detailed region of a similar soft druse from H/H AMD donor #68536. **G:** green label for Fib3; **H:** red label for CFH; **I:** merged image. Arrows point out some of the deposits showing similar levels of labeling for both antibodies. Scale bar = 50µM.

Having shown that CFH and Fib3 can interact we asked if the two proteins colocalize in macula in any examples of AMD. Immunofluorescence (IF) was used to localize Fib3 and CFH in several donor eyes including normal eyes with no histological evidence of macular degeneration, and eyes with different forms of AMD (Table 2, Fig. S2). Eyes were genotyped for the CFH 402 variant by PCR and sequencing of genomic DNA. Figure 5 shows the colocalization of CFH and Fib3 in soft drusen deposits in eyes from two different AMD donors homozygous for the CFH 402H allele (H/H). Panel A shows a section of normal human retina, with CFH localized to the choroid, Fib3 at low levels in several layers of the neural retina but no strong staining in RPE or Bruch’s membrane and no evidence of colocalization of these proteins. Panels B–E show sections from an 85 yo Caucasian male donor (#0057985) diagnosed with advanced geographic atrophy and large soft drusen visible histologically. The outer nuclear layer (ONL) is reduced due to photoreceptor loss and RPE cells are discontinuous in areas overlying the soft druse. CFH and Fib3 antibodies co-label globular clumps within the soft drusen. Substructural detail in drusen has been observed previously [12], [18]. Identical patterns of localization for CFH and Fib3 were obtained using two different primary antibodies to Fib3: the previously described mouse monoclonal raised against a C-terminal fragment of human Fib3 [39] (Fig. 5C; Fig. S3A), and a newly made antibody raised in rabbit against an N-terminal peptide of Fib3 (Fig. 5D; Fig. S3B). Panel 5E shows a section of this AMD eye containing a large hard druse which, in contrast to the soft drusen, showed no strong labeling for CFH or Fib3. For Fib3, this is consistent with previous reports [39]. In contrast to our results, others have reported the presence of CFH in hard drusen [31]. In addition to the example in panel 5E, we looked at hard drusen in other sections and again saw no strong labeling for CFH. This may reflect differences in antibody binding, such as epitope accessibility, or in classification of drusen. Anti-rhodopsin antibodies label a residual population of degenerating rod photoreceptors. Anti-glial fibrillary acidic protein labeling was associated with astrocytes and reactive Muller glia. No evidence of non-specific binding to basal deposits or drusen was observed with either of these control antibodies (Fig. 5F). Additional controls for specificity are shown in Figure S3.

**Table 2:** AMD and druse-containing eyes described in this paper.

**NDRI**	**Genotype**	**Description**	**Age/sex**
#0057985	H/H	GA dry AMD	85/M
#0068259	H/Y	“Normal”. Foveal druse	85/F
#0068280	Y/Y	Wet AMD. Scarring	85/M
#0068299	H/Y	Normal	76/M
#0068536	H/H	Dry AMD	78/F
#0068574	H/Y	Wet AMD. Scarring	91/M

GA: geographic atrophy. All eyes were from white donors.

A similar pattern of CFH and Fib3 colocalization was observed in soft drusen from a second AMD donor eye with H/H genotype. Figure 5G–I show high magnification confocal images of large soft drusen in sections of macular retina of a 78 yo Caucasian female with AMD (#0068536). The globular deposits within these soft drusen, like those of the first CFH H/H donor, show varying degrees of co-labeling for Fib3 and CFH. Thus the IF data from two different CFH 402H (H/H) donors suggest that CFH and Fib3 are colocalized rather specifically in substructures in soft drusen in an eye with geographic atrophy and an H/H genotype.

**Figure 6:**
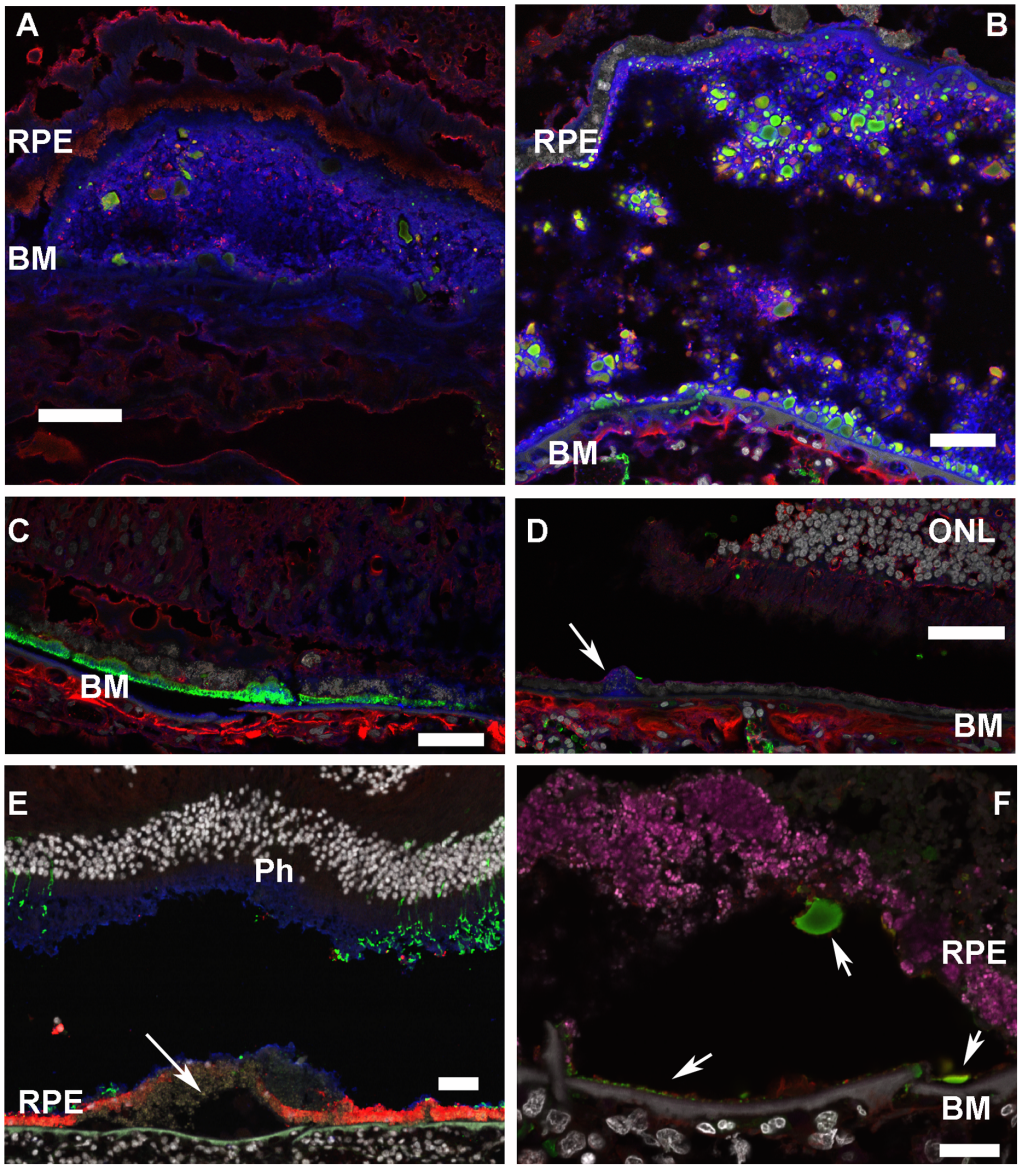
Patterns of Localization of CFH, Fib3 and cholesterol in AMD eyes vary with CFH genotype. Ph: photoreceptors; RPE: retinal pigment epithelium; BM: Bruch’s membrane. Scale bars: 50µM. **A:** Section of retina from (H/H) AMD donor #57985 labeled for CFH (red), Fib3 (rabbit polyclonal) (green), unesterified cholesterol (filipin, blue) and DAPI (white). Large soft drusen contain globules co-labeled for CFH and Fib3 embedded in cholesterol rich material. **B:** Section of retina from (H/H) AMD donor #68536 labeled as in A. This large druse also shows colocalization of CFH and Fib3 in a filipin positive region. Note that the relative intensities of CFH/Fib3 vary among deposits, leading to a range of merged colors. **C:** A region of macula from (Y/Y) donor #68280 with advanced wet AMD and fibrosis. There is a basal linear deposit strongly positive for Fib3 but lacking the globular structure and colocalization with CFH seen in H/H eyes. **D:** Section of eye from H/Y donor #68574 (wet AMD). No colocalization of CFH and Fib3. A small druse labeled with filipin (blue) is present (arrow). **E,F**: Foveal sections from H/Y “normal” donor #68259. **E:** Shows cones labeled with PNA (blue), rods labeled for rhodopsin (green), RPE labeled for RPE65 (red) and nuclei labeled with DAPI (white). Photoreceptors appear to be intact but overlie a soft druse (arrow). **F**: shows a region within the foveal druse showing punctate labeling for CFH (red) and Fib3 (green) in small adjacent deposits along Bruch’s membrane (arrow). Autofluorescence from lipofuscin granules in RPE is shown in magenta.

Figure 6A and 6B show macular drusen from both H/H donors labeled for Fib3 and CFH and also stained with the dye filipin to visualize unesterified cholesterol, a known component of drusen and basal linear deposits in AMD [6]. As shown, CFH/Fib3 positive globules in H/H eyes appear to be embedded in a cholesterol rich domain. This pattern of CFH/Fib3 colocalization was not found in other donor eyes with different CFH genotypes. Figure 6C shows sections from an eye diagnosed with AMD from an 85 yo male with Y/Y genotype (#0068280). The appearance of the eye was consistent with advanced wet AMD. The macula contained extensive regions of fibrosis and neovascularization. Fibrotic regions were positive for Fib3, but did not show the striking CFH/Fib3 positive globules seen in H/H eyes. Some regions of this Y/Y eye did contain basal linear deposits that stained intensely for Fib3 (similar to a pattern reported previously [39]), but again did not show the colocalization pattern with CFH or the soft drusen/globular structure (Fig. 6C). Another AMD eye (#0068574) with H/Y phenotype also had a different appearance from the H/H samples. As shown in Figure 6D, this eye had small cholesterol-rich drusen but no extensive colocalization of CFH and Fib3.

One more eye examined was from an 85 yo Caucasian female with no diagnosed loss of vision and an H/Y genotype (#0068259). In this eye (Fig. 6E, 6F), the peripheral retina appeared completely normal (data not shown), but the fovea showed a region of RPE dystrophy (with loss of labeling for RPE65) and a large druse. A large population of cone photoreceptors survives within this foveal region. Discrete populations of small CFH and Fib3-positive deposits are visible in this druse, adjacent to Bruch’s membrane. In contrast to the globular deposits in drusen from H/H donors, these show little evidence of CFH/Fib3 colocalization (Fig. 6F). This unusual lesion in an eye with no loss of vision might represent an early, preclinical stage in a late onset AMD. However, it has been noted that drusen, as identified by ophthalmological fundus examination, may change, even regress over time [13], [45], so the fate of a lesion such as this in a “normal” eye and its implication for development of future disease is not clear.

## Discussion

AMD is a complex disease of aging that likely results from the accumulation of multiple insults to RPE cells and their underlying Bruch’s membrane and choroid, eventually producing a crisis in which the ability of RPE cells to maintain homeostasis is exceeded. Although diverse genes and systemic risk factors are involved, the etiology of AMD focuses on the interface between the eye and its vasculature [2]. In particular, plaque-like deposits known as soft drusen accumulate in Bruch’s membrane as apparent precursors to many forms of AMD, raising the question of whether specific interactions may contribute to the formation of protein deposits and disease progression.

The common 402H variant of CFH, a key regulator of AP inflammatory processes [30], is strongly associated with increased risk of dry AMD [17], [18], [19], [20], [46]. CFH consists of multiple SCR domains with a wide variety of binding activities and it is likely that variants at residue 402 would affect the interactions of SCR7 with binding partners. Indeed, differences in binding to C-reactive protein and cell surface components have been described for the 402H/Y variants [47], [48]. Using a direct Y2H screen of CFH SCR7 against a cDNA library constructed from RPE/choroid from aged donors, a specific interaction was identified with Fib3/EFEMP1, a protein with its own associations with AMD and AMD-like disease.

There are inherited human diseases that lead to macular degeneration. In one of these, Malattia leventinese or Doyne honeycomb retinal dystrophy (ML/DHRD), Fib3/EFEMP1 is mutated (R345W) [39], [49]. This leads to formation of deposits on the basal side of the RPE and progressive loss of central visual acuity [39], [49]. In a mouse model, similar deposits within the RPE accumulate with age [50], [51], but there is no progression to RPE death. The function of Fib3 is unknown, but related proteins, Fibulins2, 4, and 5, have been implicated in TGFβ binding to ECM [52] and both Fibulin5 [53] and Fibulin6 (hemicentin 1) [54] are also loci for macular degeneration risk. Fibulins have been shown to interact with other AMD-related proteins [55], [56]. Most significantly, wild type Fib3 itself is a protein marker for AMD, accumulating at Bruch’s membrane in AMD patients [39]. Recently a copy number variant associated with the *EFEMP1/Fib3*gene in AMD patients has been described [57]. Interestingly, our Y2H experiments showed that the CFH-binding site of Fib3 was localized to its C-terminal domain, which was previously shown to be a binding site for TIMP3, another protein with macula disease associations [28], [42].

While other SCR domains could also be involved in Fib3 binding *in vivo*, the Y2H results showed that by itself SCR7 has the potential to bind this significant target protein. We next asked whether native CFH and Fib3 can interact in solution. Using media from serum-starved ARPE-19 cells as a source of native human Fib3, antibody to Fib3 co-precipitated added native CFH from solution, while antibody to CFH similarly co-precipitated Fib3.

We then used two different systems to explore the relative binding affinities of constructs for the CFH 402Y/H variants. A quantitative assay using the Y2H system showed that the H variant of SCR7 had significantly higher relative strength of binding for Fib3 than the Y variant. To further examine binding activity *in vitro*, recombinant proteins were used in ELISA. In these experiments, we chose to use a construct consisting of three SCR domains, SCR6–8 containing the disease risk domain. This strategy had been used successfully by others [37] and avoided problems in differential purification of H/Y variants of native CFH from human serum. An N-terminally tagged C-terminal construct of Fib3 was also produced to allow anchoring to the ELISA plate without blocking the C-terminal domain binding site observed in yeast. The three domain construct of CFH also bound Fib3 *in vitro* and again the construct with the disease risk H variant had a lower Kd (higher affinity). Thus a single domain construct of SCR7 in yeast and a SCR6–8 construct *in vitro* both showed similar binding to the C-terminal domain of Fib3 and in both cases the H variant had higher affinity.

The purpose of the Y2H screen was to search for potential candidate proteins from RPE/choroid that might associate with CFH and contribute to drusen formation. The screen revealed a biologically relevant candidate, Fib3. Using Y2H constructs, recombinant proteins in ELISA and Co-IP, the possibility of a CFH/Fib3 interaction was validated in different systems. However the relevance of any interaction of this type is whether it occurs *in vivo*. All these experiments led us to examine of the localization of CFH and Fib3 in normal and AMD donor eyes.

Confocal immunofluorescence localization of CFH and Fib3 in macula in several normal and AMD donor eyes gave striking results. Two donors who had dry AMD and were homozygous for the 402H allele of CFH (H/H) and showed colocalization of CFH and Fib3 in globular structures in large soft drusen in the macula. Normal eyes showed no similar localization and several examples of hard drusen (which are not strongly associated with AMD) also showed no strong labeling for either protein. Other eyes with diagnosed macular degeneration but with Y/Y genotype had a quite different appearance, with strong expression of Fib3 in fibrotic lesions and in basal linear deposits but no colocalization similar to that in H/H eyes. Intriguingly, one eye described as normal (no diagnosed AMD) with the H/Y phenotype had intact photoreceptors (consistent with no loss of vision) but also had a drusen-like lesion on the fovea that contained small punctate deposits of CFH and Fib3 but again without the globular colocalization of the H/H eyes. Whether this eye would have progressed to AMD cannot, of course, be determined but it helps illustrate of the diversity of lesions at Bruch’s membrane that may occur with genotype and stage of disease.

Both *in vitro* and *in vivo* data are consistent with association of Fib3 and CFH, particularly for the 402H variant. Although both H and Y forms of CFH showed Fib3binding in different experiments, the H form had higher affinity. It is very likely that other factors play roles in protein complex formation in drusen, perhaps enhancing the H/Y differences, but even a small increase in binding activity for the increased disease-risk variant of CFH could make a significant difference to a relatively slow, age-related accumulation of CFH/Fib3 aggregates at Bruch’s membrane. Nevertheless, similar aggregates might also form in some forms of AMD with different genetic backgrounds. The colocalized globular deposits in soft drusen in the H/H eyes in our collection could represent nucleation sites for aggregation of other components in these plaque-like deposits. Lipids have a particular association with basal deposits in AMD [6] and staining for unesterified cholesterol using the dye filipin showed that the CFH/Fib3-containing globules are embedded in cholesterol-rich domains within soft drusen. Whether this reflects a direct interaction of lipids with the protein complexes and whether other proteins are involved remain to be determined.

In the progression to dry AMD, various initial insults, perhaps related to oxidative stress or to accumulation of toxic products in aging RPE [4], [5], could lead to abnormal presentation of Fib3 at Bruch’s membrane in the macula [39]. Indeed, in our results, elevated levels of Fib3 also seem to be associated with some fibrotic lesions in eyes which appear to have advanced wet AMD and a Y/Y phenotype. As an extracellular matrix protein, Fib3 may have a general role in stress responses in damaged RPE. If CFH, which in a normal eye is localized to the choroidal side of Bruch’s membrane, also achieves access to Bruch’s membrane or to RPE, this would allow interaction with Fib3, particularly for H/H phenotypes. The formation of protein aggregates could then contribute to the growth of drusen and the development of plaque-like deposits in some forms of AMD.

Under normal circumstances, binding of CFH to a cell surface protects against complement activation [30]. It is possible that the binding of Fib3 (and perhaps other components) in soft drusen in AMD could block CFH function, thereby enhancing local complement activation. However the major complement factor C3b binding sites in CFH are remote from SCR7 [30], suggesting that Fib3 binding at this site might not by itself have a significant effect on activity. Indeed, preliminary experiments have shown no evidence that Fib3 by itself can block CFH function in a simple C3b cleavage assay (unpublished). Alternatively, the sequestration of CFH in drusen aggregates might block its ability to regulate the alternative pathway locally. Another possibility is that CFH/Fib3-containing complexes may have a structural role in initiating the accretion of larger multi-component, plaque-like aggregates in Bruch’s membrane. Such abnormal aggregates could compromise function of already stressed RPE cells by disrupting normal interactions with choroid, including transport of metabolites and responses to growth factors. Indeed, a role for amyloid-like deposits in AMD has been implicated in a mouse model [58]. As in fibrotic diseases and atherosclerosis [8], [10] formation of plaque-like deposits could themselves trigger inflammatory responses [59].

CFH/Fib3 binding may be just one link in a complex chain of events that progresses towards RPE pathology, photoreceptor death and blindness in some forms of AMD, but it offers the interesting possibility of a new target for therapeutic intervention that could perhaps slow the progression of this major cause of vision loss in the aging human population.

## Supporting Information

Figure S1 **Specificity of antibodies to Fib3.** Mouse monoclonal (m) and rabbit polyclonal peptide (r) antibodies to human Fib3 were tested in Western blots of GST-Fib3 fusion protein and ARPE-19 cell conditioned medium. Identical results were obtained. The same antibodies also give identical results in IF experiments.
(TIF)

Figure S2 **Localization and histology of Cryosections of donor eyes.** A) White rectangle indicates the region cut for sectioning from each donor eye. A portion of optic nerve head was included for orientation. Fundus image NEI #EDA06. B) H&E staining of cryosection from donor eye #57985 in region of large soft drusen. INL: inner nuclear layer; ONL: outer nuclear layer; RPE: retinal pigment epithelium; BM: Bruch’s membrane; CH: choroid; SD: soft druse. Magnification 10X. C) Cryosection from donor eye #68536 stained and labeled as in B). Magnification 4X. D) Cryosection from donor eye #68280 stained and labeled as in B). Magnification 10X.
(TIF)

Figure S3 **Specificity of antibodies used in Immunofluorescence.** Cryosection from eye #57985 labeled with A) mouse anti-Fib3 (green); B) rabbit anti-Fib3 (blue); C) goat anti-CFH (red) antibodies. DAPI: white. Both antibodies to Fib3 give identical patterns and co-localize with CFH in the druse. INL: inner nuclear layer: ONL: outer nuclear layer (severely degenerated). D,E) Cryosections with no primary antibody, labeled only with secondary antibodies: D: Alexa 488 donkey anti-rabbit (green) and E) Alexa 555 donkey anti-goat (red). Only autofluorescence from RPE and Bruch’s membrane is apparent in green or red channels.
(TIF)
