# Supplementary figures and images for: Interaction of Complement Factor H and Fibulin3 in Age-Related Macular Degeneration
Source: PLoS One. 2013 Jun 28;8(6):e68088. doi: 10.1371/journal.pone.0068088 (PMC3696004; doi:10.1371/journal.pone.0068088)

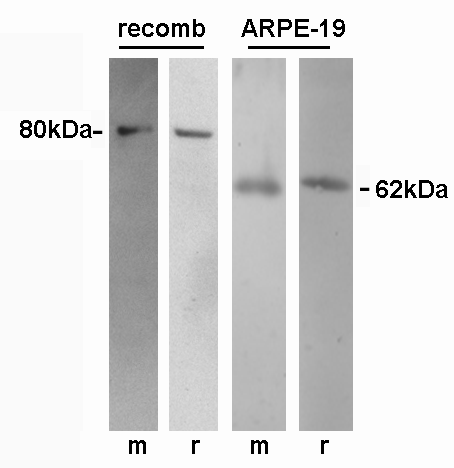

Supplement: Figure S1 — Specificity of antibodies to Fib3. Mouse monoclonal (m) and rabbit polyclonal peptide (r) antibodies to human Fib3 were tested in Western blots of GST-Fib3 fusion protein and ARPE-19 cell conditioned medium. Identical results were obtained. The same antibodies also give identical results in IF experiments. (TIF) [file pone.0068088.s001.tif]

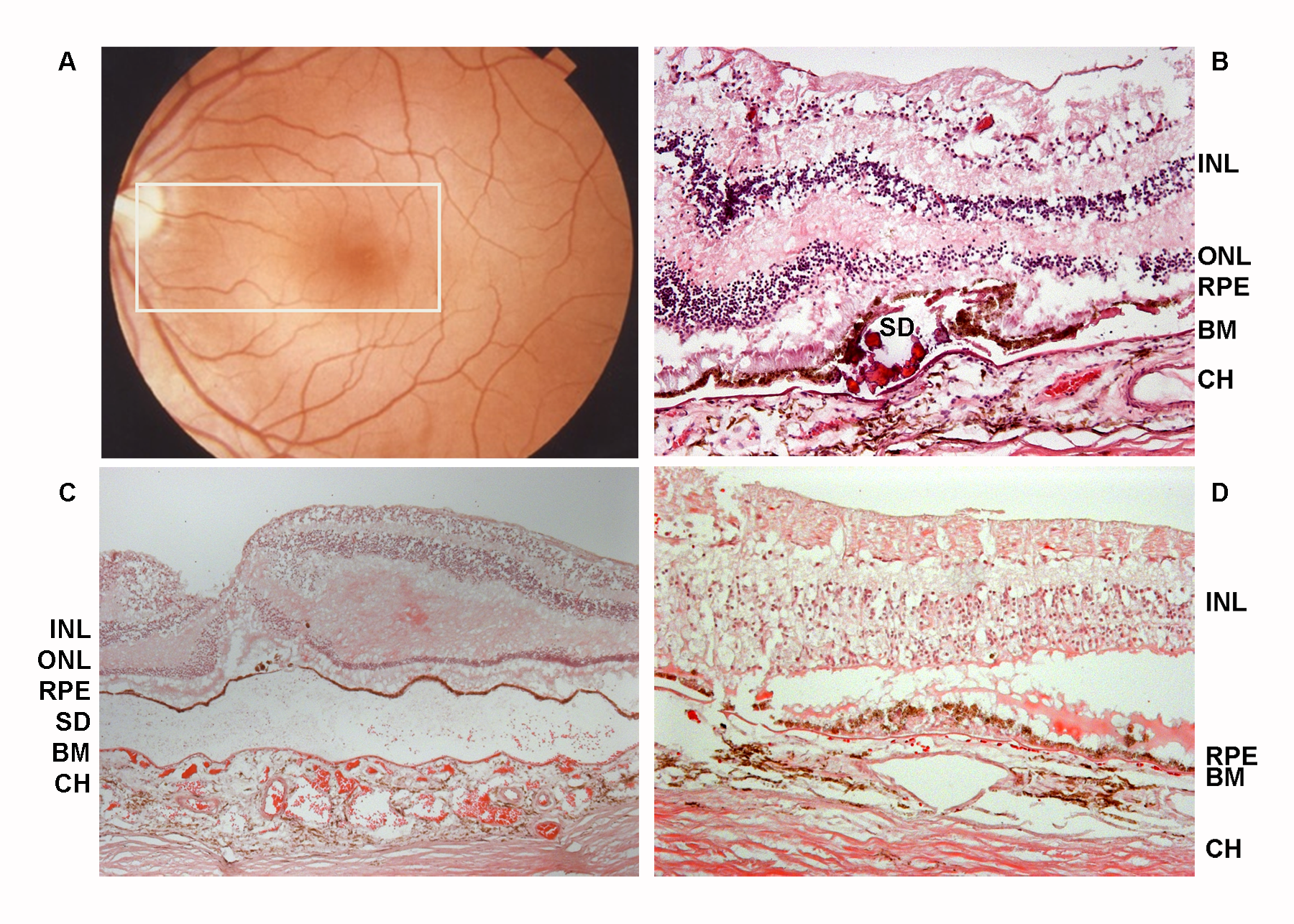

Supplement: Figure S2 — Localization and histology of Cryosections of donor eyes. A) White rectangle indicates the region cut for sectioning from each donor eye. A portion of optic nerve head was included for orientation. Fundus image NEI #EDA06. B) H&E staining of cryosection from donor eye #57985 in region of large soft drusen. INL: inner nuclear layer; ONL: outer nuclear layer; RPE: retinal pigment epithelium; BM: Bruch’s membrane; CH: choroid; SD: soft druse. Magnification 10X. C) Cryosection from donor eye #68536 stained and labeled as in B). Magnification 4X. D) Cryosection from donor eye #68280 stained and labeled as in B). Magnification 10X. (TIF) [file pone.0068088.s002.tif]

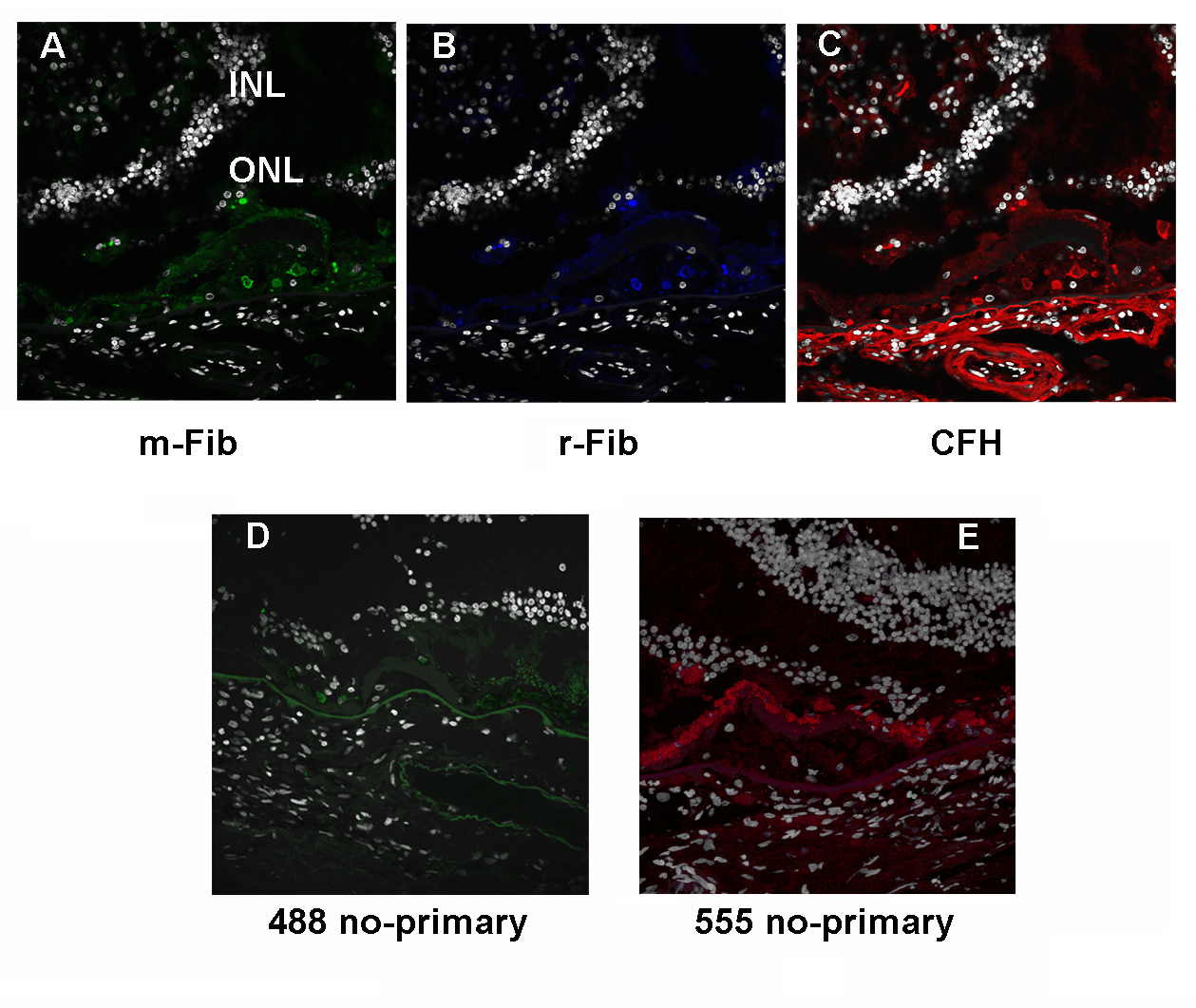

Supplement: Figure S3 — Specificity of antibodies used in Immunofluorescence. Cryosection from eye #57985 labeled with A) mouse anti-Fib3 (green); B) rabbit anti-Fib3 (blue); C) goat anti-CFH (red) antibodies. DAPI: white. Both antibodies to Fib3 give identical patterns and co-localize with CFH in the druse. INL: inner nuclear layer: ONL: outer nuclear layer (severely degenerated). D,E) Cryosections with no primary antibody, labeled only with secondary antibodies: D: Alexa 488 donkey anti-rabbit (green) and E) Alexa 555 donkey anti-goat (red). Only autofluorescence from RPE and Bruch’s membrane is apparent in green or red channels. (TIF) [file pone.0068088.s003.tif]
